# RPEL Proteins Are the Molecular Targets for CCG-1423, an Inhibitor of Rho Signaling

**DOI:** 10.1371/journal.pone.0089016

**Published:** 2014-02-18

**Authors:** Ken’ichiro Hayashi, Bunta Watanabe, Yoshiaki Nakagawa, Saki Minami, Tsuyoshi Morita

**Affiliations:** 1 Department of Neuroscience (D13), Osaka University Graduate School of Medicine, Osaka, Japan; 2 Institute for Chemical Research, Kyoto University, Kyoto, Japan; 3 Division of Applied Life Sciences, Graduate School of Agriculture, Kyoto University, Kyoto, Japan; Hokkaido University, Japan

## Abstract

Epithelial–msenchymal transition (EMT) is closely associated with cancer and tissue fibrosis. The nuclear accumulation of myocardin-related transcription factor A (MRTF-A/MAL/MKL1) plays a vital role in EMT. In various cells treated with CCG-1423, a novel inhibitor of Rho signaling, the nuclear accumulation of MRTF-A is inhibited. However, the molecular target of this inhibitor has not yet been identified. In this study, we investigated the mechanism of this effect of CCG-1423. The interaction between MRTF-A and importin α/β1 was inhibited by CCG-1423, but monomeric G-actin binding to MRTF-A was not inhibited. We coupled Sepharose with CCG-1423 (CCG-1423 Sepharose) to investigate this mechanism. A pull-down assay using CCG-1423 Sepharose revealed the direct binding of CCG-1423 to MRTF-A. Furthermore, we found that the N-terminal basic domain (NB) of MRTF-A, which acts as a functional nuclear localization signal (NLS) of MRTF-A, was the binding site for CCG-1423. G-actin did not bind to CCG-1423 Sepharose, but the interaction between MRTF-A and CCG-1423 Sepharose was reduced in the presence of G-actin. We attribute this result to the high binding affinity of MRTF-A for G-actin and the proximity of NB to G-actin-binding sites (RPEL motifs). Therefore, when MRTF-A forms a complex with G-actin, the binding of CCG-1423 to NB is expected to be blocked. NF-E2 related factor 2, which contains three distinct basic amino acid-rich NLSs, did not bind to CCG-1423 Sepharose, but other RPEL-containing proteins such as MRTF-B, myocardin, and Phactr1 bound to CCG-1423 Sepharose. These results suggest that the specific binding of CCG-1423 to the NLSs of RPEL-containing proteins. Our proposal to explain the inhibitory action of CCG-1423 is as follows: When the G-actin pool is depleted, CCG-1423 binds specifically to the NLS of MRTF-A/B and prevents the interaction between MRTF-A/B and importin α/β1, resulting in inhibition of the nuclear import of MRTF-A/B.

## Introduction

Myocardin (Mycd) family members are specific coactivators of serum response factor (SRF) and play a critical role in the activation of SRF-mediated transcription [1], [2]. They include Mycd, myocardin-related transcription factor A (MRTF-A/MAL/MKL1), and MRTF-B (MAL16/MKL2). Although Mycd is expressed specifically in cardiac and smooth muscles [3], [4], MRTF-A/B are expressed in a wide variety of cells and tissues [2], [5], [6]. Mycd is constitutively located in the nucleus [3], whereas MRTF-A/B reside primarily in the cytoplasm and transiently translocate to the nucleus in response to Rho activation [2], [7], [8]. MRTF-A/B participate in various biological processes and cell functions [9], [10], [11], [12] and play a critical role in extracellular stimulation-induced epithelial–mesenchymal transition (EMT), which arises from the enhanced expression of several cytoskeletal proteins triggered by Rho activation [2], [13]. This process is closely associated with cancer progression and metastasis [14] and tissue fibrosis [15], [16].

We have recently shown that importin α/β1 and CRM1 (exportin1/XPO1) regulate the nuclear import and export, respectively, of Mycd family members [17], [18]. The N-terminal basic domain (NB) of Mycd family members (also known as B2 [2]), which is located between the second and third G-actin-binding RPEL motifs and is conserved among all members of Mycd family, is a binding site for importin α/β1 and functions as a nuclear localization signal (NLS). Actin dynamics do not affect the interaction between Mycd and importin α/β1 and the nuclear localization of Mycd, whereas G-actin significantly suppresses the interaction between MRTF-A/B and importin α/β1 and affects the nuclear import of MRTF-A/B [17], [19]. In the presence of G-actin, MRTF-A/B preferentially form a complex with G-actin owing to their high binding affinity for G-actin, resulting in blocking access of importin α/β1 to NB. The nuclear import of Phactr1, one of the other RPEL-containing proteins, is similarly regulated [20]. Phactr1 contains four RPEL motifs and two basic amino acid-rich NLSs, which are in close proximity to the RPEL motifs. Phactr1 nuclear accumulation is mediated by importin α/β1. In resting cells, actin binding to the three C-terminal RPEL motifs inhibits the nuclear accumulation of Phactr1. This inhibition is due to the competitive binding of G-actin and importin α/β1 to NLSs associated with the N- and C-terminal RPEL motifs.

CCG-1423 was originally identified as an inhibitor of RhoA signaling [21]. Although CCG-1423 has been reported to block the nuclear accumulation of MRTF-A [22], [23], the molecular mechanism is yet to be determined. Based on the cumulative evidence, we speculated that CCG-1423 directly inhibits MRTF-A binding to importin α/β1. In this study, we addressed this hypothesis and identified the inhibitory mechanism of this small molecule. These findings suggest a possible strategy(s) for anti-EMT drug discovery.

## Materials and Methods

### Reagents and Antibodies

CCG-1423, anti-NF-E2 related factor 2 (Nrf2), and anti-importin β1 antibodies were purchased from Santa Cruz Biotechnology (Santa Cruz, CA). Jasplakinolide (Jasp) and latrunculin B (LatB) were purchased ftom Caymen Chemical (Ann Arbor, MI). Other antibodies used in this study were anti-Flag M2 affinity gel (Sigma, St. Louis, MO), anti-HA affinity matrix and anti-HA (3F10) antibody (Roche Applied Science, Mannheim, Germany), and anti-DYKDDDDK (anti-Flag) antibody (Trans Genic, Kobe, Japan). Secondary antibodies and phalloidin were conjugated to Alexa 568 (Molecular Probes, Eugene, OR). We produced a polyclonal antibody against MRTF-A. In brief, a peptide consisting of amino acids 714–728 of mouse MRTF-A (MAL met; GenBank accession number: BC050941.1) was injected into a rabbit, and the antiserum was subjected to affinity purification.

### Plasmids

The construction of the plasmids used in this study, except for the mouse Nrf2 and rat Phactr1 expression plasmids, is described elsewhere [17], [18], [24]. In brief, each of cDNAs of mouse Mycd family members, mouse Nrf2 (NCBI Reference Sequence: NM_010902.3), and rat Phactr1 (NCBI Reference Sequence: NM_214457.2) were amplified by reverse transcription PCR and inserted into a mammalian expression plasmid, pCS2+, with a Flag tag at the N-termini. The sequences were confirmed.

### Cell Culture and Immunocytochemistry

NIH3T3 cells were cultured in Dulbecco’s modified Eagle’s medium supplemented with 10% fetal calf serum. Transfection of the indicated plasmids was performed using Trans IT-LT1 (PanVera Corporation, Madison, WI). The transfected cells were then cultured under the indicated conditions for 24 h. Immunocytochemistry was performed according to previously reported procedures [18]. The cells were incubated with phalloidin conjugated to Alexa Fluor 568 or the indicated primary antibodies followed by the specified secondary antibodies with Hoechst 33258. Fluorescent images were collected with the aid of a Biorevo BZ-9000 fluorescence microscope (Keyence, Osaka, Japan). The expression patterns of MRTF-A were categorized into three groups: nuclear-specific localization (N); diffuse distribution in the nucleus and the cytoplasm (NC), defined as equivalent immunostaining intensities of the target molecules in the cytoplasm and nucleus; and cytoplasmic localization (C). In each experiment, 100–200 cells were examined. The proportion of cells exhibiting the respective expression patterns is presented.

### Promoter Assay

NIH3T3 cells were transfected with the indicated plasmids and cultured for 24 h. For further 20 h, the cells were cultured under serum-starved conditions and were re-stimulated with serum for 4 h. Cell extracts prepared using a passive lysis buffer (Promega) were subjected to luciferase assay with a luciferase assay kit (Promega). Relative promoter activity was expressed in luminescence units normalized to the β-galactosidase activity of pSVβ-gal in the cell extracts. These assays were performed in triplicate and were repeated three times.

### Protein–protein Interaction in vitro

All of the proteins used in this analysis were prepared using the TNT SP6 High-Yield Expression System based on an optimized wheat germ extract (Promega). We preliminarily confirmed that there was no significant protein cross-reaction between any of the antibodies against the indicated tag peptides, nuclear import proteins, and wheat germ extract for in vitro-translation and evaluated the expression levels of the respective in vitro-translated proteins by immunoblotting (IB) using previously specified antibodies [17], [18]. The composition of the immunoprecipitation (IP) buffer in this study was as follows: 20 mM Tris-HCl (pH 7.5), 0.5% Nonidet P-40, 150 mM NaCl, 1 mM EDTA, 50 mM NaF, 10 mM β-glycerophosphate, and proteinase inhibitors [complete Mini (Roche Applied Science)]). The IP buffer mixtures (total 500 µl) containing Flag-tagged MRTF-A proteins (15 or 30 µl), defined amounts of the indicated proteins [HA-tagged importin α1 protein, 10 µl; importin β1 protein 10 µl; β-actin R62D (unpolymerized mutant) protein, 20 µl], and CCG-1423 (10 µM) or vehicle [dimethyl sulfoxide (DMSO)] were subjected to IP analyses as previously described [17]. Proteins in the immunoprecipitates were detected by IB. Target proteins were detected with a SuperSignal chemiluminescence detection kit (Pierce, Rockford, IL). For IB analysis, 3.3% of the input proteins and 22.2% of the IP proteins were loaded on the input and IP lanes, respectively. Quantification of the respective IB signals’ intensities was performed with the NIH ImageJ software. These interaction analyses were repeated three times.

### Protein–protein Interaction in Cultured Cells

NIH3T3 cells were transfected with the expression plasmids for Flag-MRTF-A, HA-importin α1, and importin β1. The transfected cells were cultured under serum-stimulated conditions for 30 h. For the final 16 h, the cells were cultured in the presence of either 10 µM CCG-1423 or vehicle (DMSO), and then were re-stimulated with fresh serum for 15 min. Whole cell extracts were prepared by incubation with the IP buffer, followed by sonication. The whole cell extracts thus obtained were subjected to IP/IB analyses as described above.

### Sepharose Covalently Coupled with CCG-1423

Preparation of CCG-1423 affinity Sepharose was based on previously reported methods [25], [26], [27]. The structure of the photoaffinity linker was characterized by ^1^H NMR, recorded on Bruker AVANCEIII 400 (400 MHz for ^1^H) or JEOL JNM-AL300 (300 MHz for ^1^H) spectrometers.

### Confirmation of Direct Binding of CCG-1423 to RPEL Proteins

Direct binding of CCG-1423 to each of Mycd family members and Phactr1 was examined by pull-down assay using CCG-1423 Sepharose. The indicated in vitro-translated proteins were purified using anti-Flag M2 affinity gel or anti-HA affinity matrix and were used as inputs. The IP buffer (total 400 µl) containing the indicated protein(s) (300 ng), 0.005% bovine serum albumin, CCG-1423 Sepharose or control Sepharose without CCG-1423 coupling (bed volume 25 µl), and free CCG-1423 (10 µM) or DMSO were incubated at 4°C for 2 h with gentle shaking. After washing with the IP buffer, followed by washing with phosphate-buffered saline, the pull-down proteins were detected by IB. Detection and quantification of target proteins were performed as described earlier. For IB analysis, 20% of the input proteins and 22% of the pull-down proteins were loaded on the input and the pull-down lanes, respectively. These interaction analyses were repeated three times.

### Binding Assay of MRTF-A/B and Phactr1 to CCG-1423 Sepharose Using NIH3T3 Cell Whole Extracts

NIH3T3 cells were transfected with each of the expression plasmids for Flag-MRTF-A, Flag-MRTF-B, and Flag-Phactr1 and were cultured under serum-stimulated conditions for 30 h. For the final 16 h, they were cultured under either serum-stimulated or serum-starved conditions. The serum-starved cells were further cultured for 10 min in the presence of 2 µM of LatB (an inhibitor of actin polymerization). The serum-stimulated cells were further re-stimulated with fresh serum for 15 min. Whole cell extracts were prepared by incubation with the IP buffer containing either 1 µM of Jasp (a stabilizer of F-actin) for serum-stimulated cells or 2 µM LatB for serum-starved cells followed by sonication. The resulting whole cell extracts were subjected to the binding assay using CCG-1423 Sepharose as described earlier.

### Statistical Analysis

All graphs show means and standard errors. Statistical analysis was performed using Student’s t-test.

## Results

### CCG-1423 Treatment Inhibits Serum-induced Nuclear Accumulation of MRTF-A

We examined the effect of CCG-1423 on the subcellular localization of exogenously expressed Flag-MRTF-A in NIH3T3 cells under serum-starved and serum-stimulated conditions (Figure S1A, B). In majority (51.9±7.7%) of the cells expressing Flag-MRTF-A under serum-starved conditions, the protein was primarily observed in the cytoplasm. In contrast, in a large proportion (66.4±0.7%) of serum-stimulated cells, Flag-MRTF-A protein accumulated primarily in the nucleus. CCG-1423 treatment markedly reduced (17.5±1.6%) the proportion of cells showing the nuclear accumulation of the protein. In majority (53.2±5.0%) of the cells treated with CCG-1423, the protein was evenly distributed in the cytoplasm and nucleus. In correspondence with these changes, the protein’s transactivation ability for the SM22α promoter also reduced in CCG-1423-treated cells (Figure S1C). Similar to that of exogenously expressed Flag-MRTF-A, serum-induced nuclear accumulation of endogenous MRTF-A was inhibited by CCG-1423 (Figure S1D, E). These results suggest that CCG-1423 treatment inhibits serum-induced nuclear import of MRTF-A.

### CCG-1423 Inhibits MRTF-A Binding to Importin α/β1

**Figure 1:**
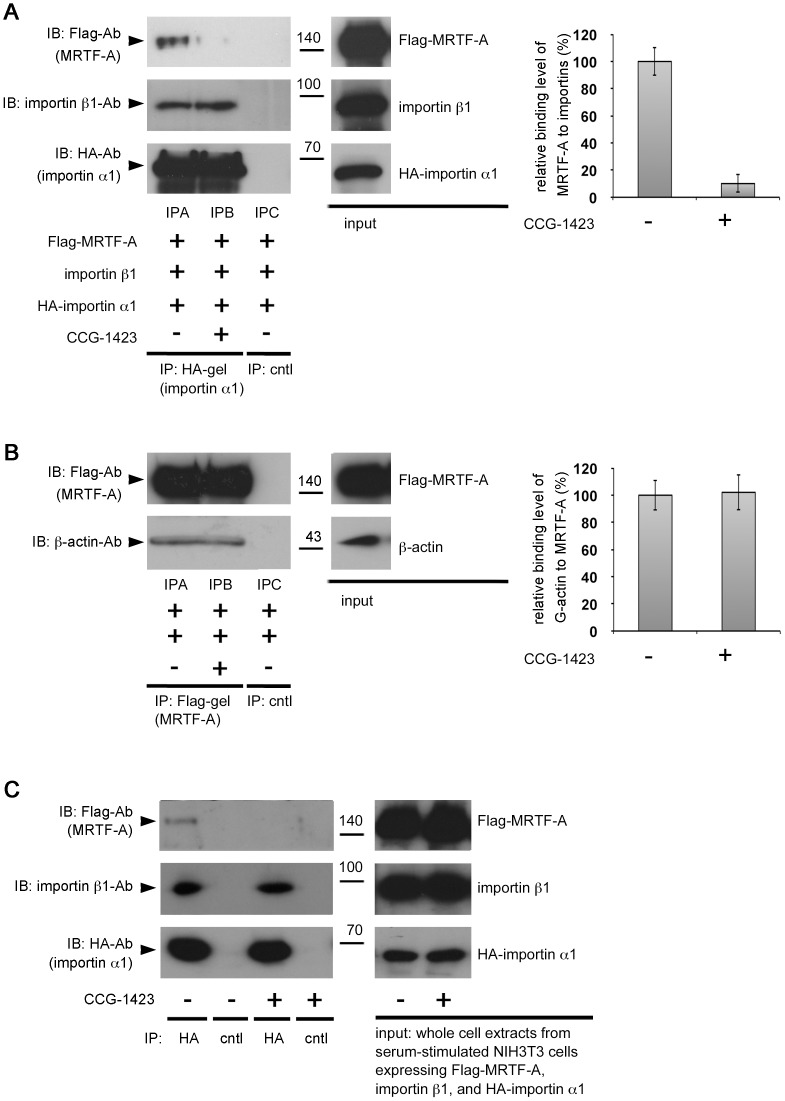
Effects of CCG-1423 on the in vitro interaction between MRTF-A and importin α/β1. Mixtures of in-translated HA-importin α1, importin β1, and Flag-MRTF-A proteins (A) or β-actin R62D (G-actin) and Flag-MRTF-A proteins (B) were immunoprecipitated with a control gel, anti-HA-affinity matrix, or anti-Flag M2 affinity gel in the presence of either CCG-1423 (+) or vehicle (DMSO; −), and the resulting immunoprecipitates were analyzed by immunoblotting (IB) with the indicated antibodies. Positions of molecular weight markers are indicated on the side of IB panels in kilodaltons (left columns). Control experiments with the control gel (cntl) showed no significant signals on IB (lanes IPC). The respective immunoprecipitation (IP)/IB signal intensities were quantified as described in Materials and Methods (right columns). The percentage values indicate the relative levels of MRTF-A binding to importin α1/β1 (A) or G-actin binding to MRTF-A (B) normalized by the binding of MRTF-A or G-actin in the absence of CCG-1423, which were set at 100% (mean ± s.e.m of the results from three independent experiments). (C) Inhibitory effect of CCG-1423 on the interaction between MRTF-A and importin α/β1 in cultured cells. NIH3T3 cells were transfected with the expression plasmids as described in Materials and Methods. Whole cell extracts from the cells re-stimulated with serum were subjected to IP/IB analysis as described earlier. Representative data are shown (n  = 3).

We speculated that CCG-1423 directly inhibits the nuclear import of MRTF-A. To test this hypothesis, we examined the effect of CCG-1423 on the interaction between MRTF-A and importin α/β1 in vitro. In the presence of CCG-1423, the binding of MRTF-A to importin α/β1 markedly reduced, but the formation of importin αβ1 heterodimer did not reduce (Figure 1A, lane IPB). G-actin binding to MRTF-A was also unaffected by CCG-1423 (Figure 1B, lane IPB). These results suggest a possibility that CCG-1423 binds directly to MRTF-A and prevents the interaction between MRTF-A and importin α/β1 but does not impair the function of RPEL motifs as the G-actin-binding sites. To confirm the in vitro results, we examined the inhibitory effect of CCG-1423 on the interaction between MRTF-A and importin α/β1 in culture cells. This interaction was detected in DMSO-treated cells, but was inhibited in CCG-1423-treated cells (Figure 1C).

### Direct Binding of CCG-1423 to MRTF-A Mediated by the NB

**Figure 2:**
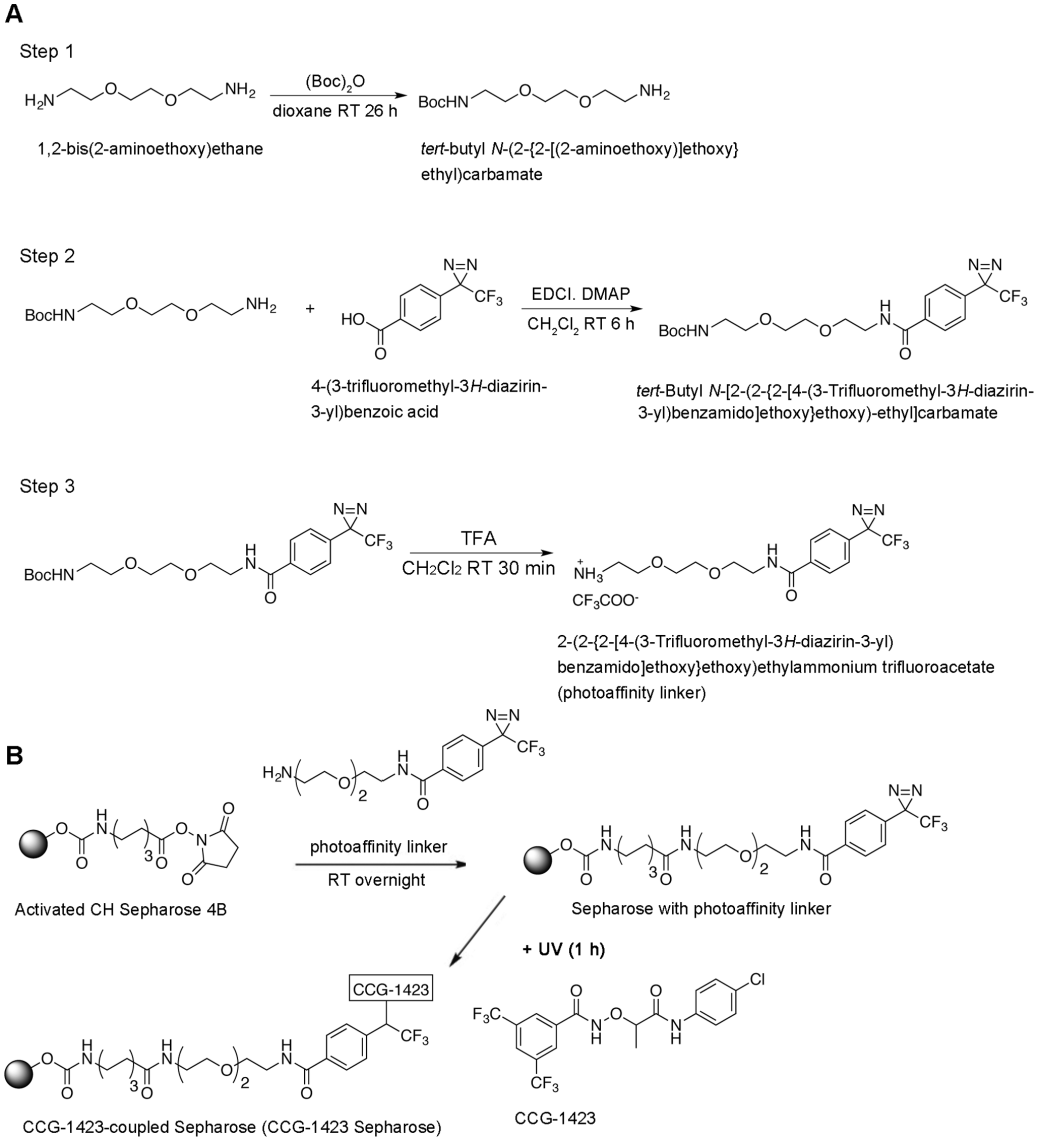
Preparation of CCG-1423 Sepharose. (A) Synthesis of the photoaffinity linker, 2-(2-{2-[4-(3-trifluoromethyl-3*H*-diazirin-3-yl)benzamido]ethoxy}ethoxy)ethylammonium trifluoroacetate. The photoaffinity linker was synthesized by the indicated three-step reactions. The steps were based on the following methods: step 1 [24] and steps 2 and 3 [25]. Reagents needed for the respective reactions are indicated by their abbreviations (above the arrows) and are as follows: (Boc)_2_O, di-*tert*-butyl dicarbonate; EDCI, 1-ethyl-3-(3-dimethylaminopropyl) carbodiimide hydrochloride, DMAP, 4-(dimethylamino)pyridine; TFA, trifluoroacetic acid. Solvents and reaction time for the respective reactions are indicated (below the arrows). The structures of the respective synthesized products were characterized by NMR. (B) Crosslinking of CCG-1423 to Sepharose with the photoaffinity linker. This step was performed according to the method published by McIntyre et al. [26]. Activated CH Sepharose 4B beads were coupled with the photoaffinity linker, and the beads were treated with 1 M ethanolamine (pH 11) to block the remaining reactive groups. The Sepharose beads with photoaffinity linker were agitated with 50 mM Tris-HCl (pH 7.4) buffer containing 0.1 mM CCG-1423, and then were exposed to UV light for 1 h. CCG-1423 was randomly coupled with the photoaffinity linkers on Sepharose by UV irradiation. The CCG-1423 Sepharose was washed with methanol and dried.

**Figure 3:**
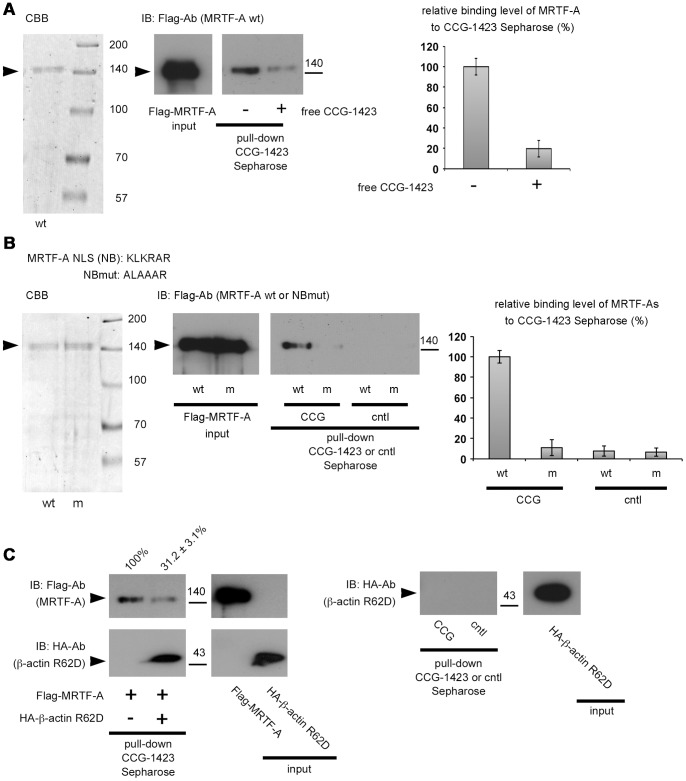
Direct binding of CCG-1423 to MRTF-A mediated by NB. (A and B) Examination of the binding of MRTF-A to CCG-1423 Sepharose. Coomassie brilliant blue (CBB) staining of purified proteins used in these assays are as follows: Flag-MRTF-As [wild-type (wt) and NBmut (m)] (A and B, left columns). (C) Investigations of the binding of Flag-MRTF-A to CCG-1423 Sepharose in the presence of G-actin and the binding of G-actin to CCG-1423 Sepharose. Pull-down assays with CCG-1423 Sepharose were performed using purified Flag-MRTF-A and/or HA-β-actin R62D (G-actin) proteins as inputs. Detailed procedures are described in Materials and Methods. The proteins bound to CCG-Sepharose (CCG) or control Sepharose (cntl) were analyzed by IB with the indicated antibodies (A and B, middle columns and C). The respective pull-down/IB signal intensities were quantified (A and B, right graphs and C, the percentage values on the top of pull-down column). The percentage values indicate the relative levels of MRTF-A binding to CCG-1423 Sepharose or control Sepharose normalized by the binding of MRTF-A in the absence of free CCG-1423 (A), the binding of wild-type MRTF-A to CCG-1423 Sepharose (B), or the binding of MRTF-A in the absence of G-actin (C), which was set at 100% (mean ± s.e.m of the results from three independent experiments).

We covalently coupled Sepharose with CCG-1423 (CCG-1423 Sepharose) using a photo-crosslinking agent (Figure 2) and performed a pull-down assay using the CCG-1423 Sepharose to examine MRTF-A binding to CCG-1423 (Figure 3A, B). Because MRTF-A is associated with various proteins including G-actin, SRF, Smad, and other protein factors in cells, these protein factors may affect the interaction between MRTF-A and CCG-1423 Sepharose. In these assays, to rule out this possibility, in vitro-translated Flag-tagged proteins were purified using an anti-Flag M2 affinity gel and were used as inputs (Figure 3A, B, left columns). Wild-type MRTF-A protein clearly bound to CCG-1423 Sepharose, but such binding was severely reduced by free CCG-1423 (20.6±6.3% of the binding level in the absence of free CCG-1423) (Figure 3A, middle and right columns). However, this protein did not bind to a control Sepharose without CCG-1423 coupling (Figure 3B, middle and right columns). An MRTF-A protein with mutation in NB (MRTF-A NBmut), in which the NB sequence KLKRAR was mutated to ALAAAR, exhibited a low binding level (11.2±8.5% of the wild-type protein level) (Figure 3B, middle and right columns). These results strongly suggest that CCG-1423 binds specifically and directly to MRTF-A under mediation by NB. The basic amino acids in the NB sequence (Figure 3B) play a critical role in CCG-1423 binding to NB.

Because NB is in close proximity to the G-actin-binding RPEL motifs (Figure 7), we predicted that CCG-1423 binding to NB competes with G-actin binding to RPEL motifs. We addressed this possibility using purified MRTF-A and β-actin R62D (G-actin) proteins. In the presence of G-actin, MRTF-A binding to CCG-1423 Sepharose reduced, but G-actin was clearly detected in the bound fraction (Figure 3C, left column). G-actin did not bind solely to CCG-1423 Sepharose (Figure 3C, right column). Taken together with the data shown in Figure 1B, CCG-1423 does not inhibit G-actin binding to MRTF-A, suggesting that in the presence of G-actin, MRTF-A preferentially forms a complex with G-actin because of its high binding affinity for G-actin and results in inhibition of CCG-1423 binding to MRTF-A. Thus, G-actin-free MRTF-A rather than MRTF-A associated with G-actin is the more likely CCG-1423 target protein.

**Figure 4:**
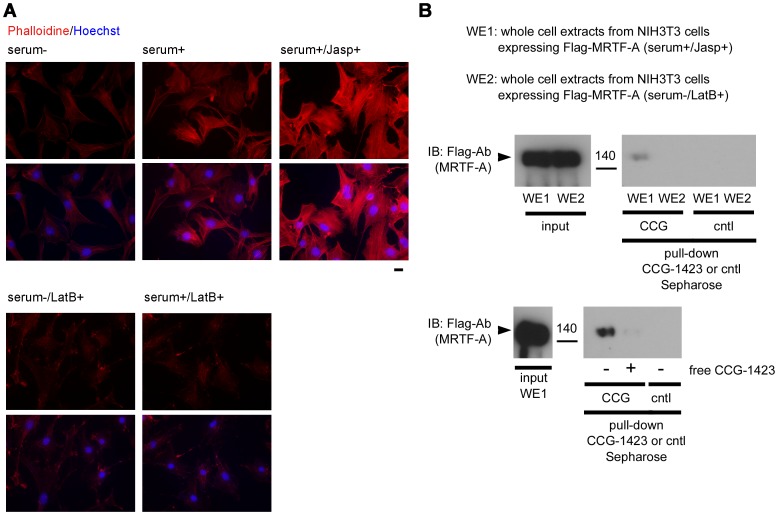
Effects of actin dynamics on MRTF-A binding to CCG-1423 Sepharose. (A) Imaging of F-actin in NIH3T3 cells by staining with phalloidin conjugated to Alexa Fluor 568. NIH3T3 cells were cultured under serum-stimulated conditions. For the final 16 h, they were cultured under either serum-starved (serum−) or serum-stimulated (serum+) conditions. The cells were further incubated with either 50 nM of Jasp (Jasp+) or 2 µM LatB (LatB+) for 10 min. Bars  = 20 µm. (B) Whole cell extracts (WE1 and WE2) were prepared from NIH3T3 cells expressing Flag-MRTF-A under either serum-stimulated or serum-starved conditions. Brief explanations of the respective whole cell extracts are given in the upper panel; WE1 from serum-stimulated cells contains Jasp (Jasp+) and WE2 from serum-starved cells contains LatB (LatB+). The details of whole cell extract preparation are described in Materials and Methods. These whole cell extracts were subjected to pull-down assay using CCG-1423 Sepharose in the absence or presence of free CCG-1423 (10 µM). The proteins bound to CCG-Sepharose (CCG) or control Sepharose (cntl) were analyzed by IB with anti-Flag antibody.

**Figure 5:**
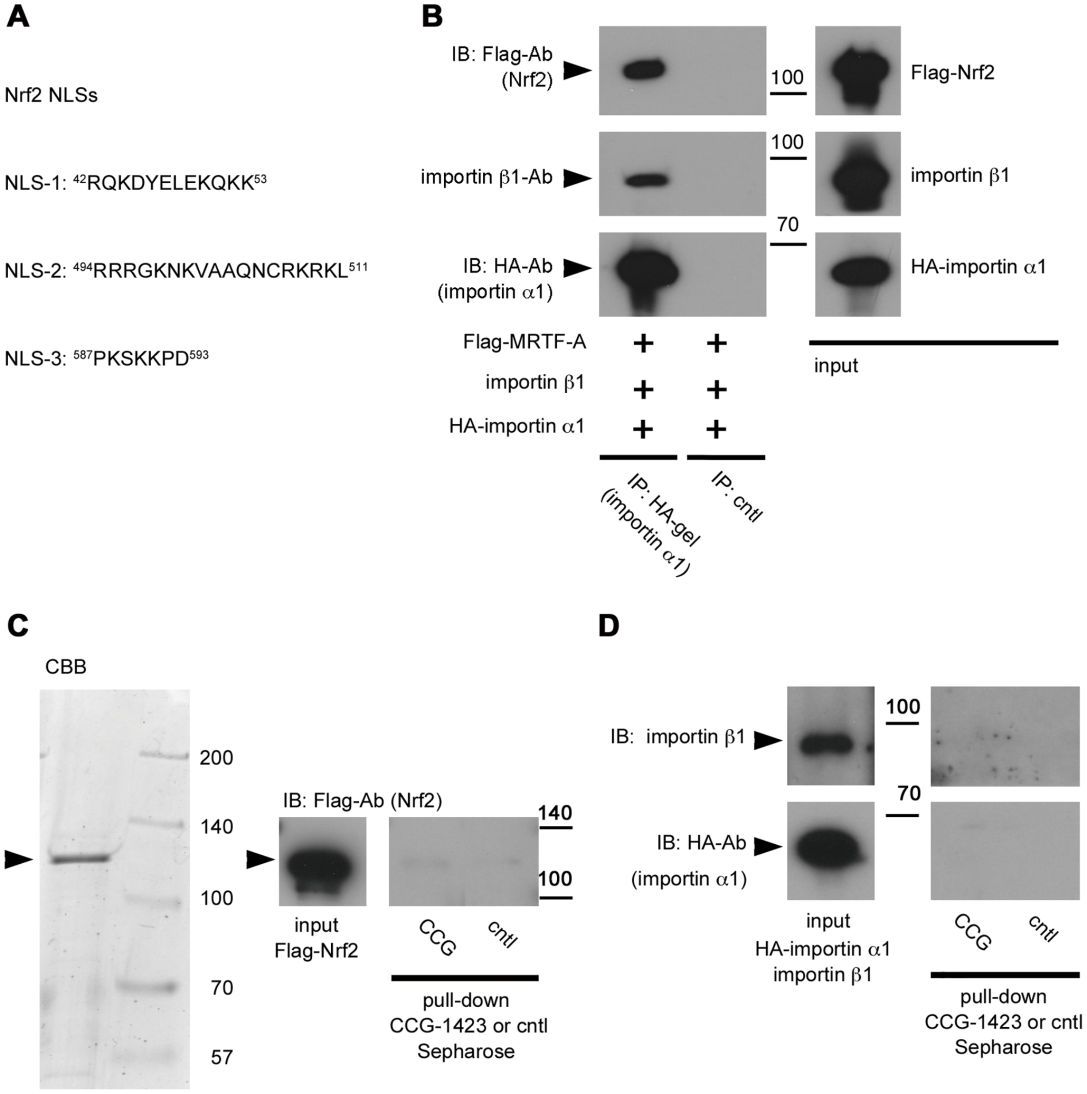
Binding specificity of CCG-1423. (A) The sequences of Nrf2 NLSs are aligned. (B) In vitro interaction between Nrf2 and importin α/β1. Mixtures of in vitro-translated HA-importin α1, importin β1, and Flag-Nrf2 proteins were immunoprecipitated with a control gel (cntl) or anti-HA-affinity matrix, and the resulting immunoprecipitates were analyzed by IB with the indicated antibodies. Positions of molecular weight markers are indicated on the side of IB panels in kilodalton. (C) Examination of the binding of purified Flag-Nrf2 to CCG-1423 Sepharose. Procedures for the pull-down assays are similar to those described in the legend for Figure 3. Representative data are shown (n  = 3). (D) Examination of the binding of importin α/β1 to CCG-1423 Sepharose. A mixture of in vitro-translated HA-importin α1 and importin β1 proteins was incubated for 1 h on ice to form a heterodimeric complex. The complex thus obtained was purified using anti-HA-affinity matrix and was used as input. The pull-down assays were performed as described in the legend for Figure 3. Representative data are shown (n  = 3).

**Figure 6:**
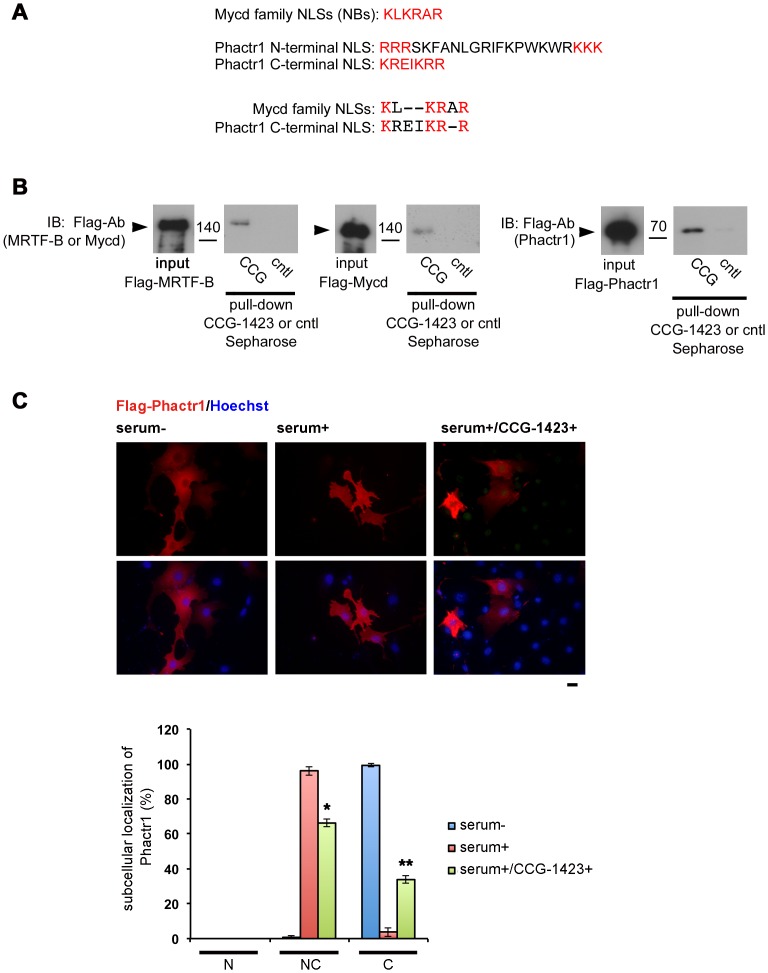
Direct binding of CCG-1423 to other Mycd family members and Phactr1. (A) The sequences of NLSs of Mycd family members and Phactr1 are aligned. Amino acids indicated with red letters play a critical role as an NLS of each RPEL-containing protein. The sequence of NLS of all members of the Mycd family is completely conserved. Phactr1 N-terminal NLS abuts the first RPEL motif, and Phactr1 C-terminal NLS is located between the third and fourth RPEL motifs. (B) Examination of the binding of purified Flag-MRTF-B, Flag-Mycd, or Flag-Phactr1 to CCG-1423 Sepharose. The pull-down assays were performed as described in the legend for Figure 3. (C) Effects of CCG-1423 on the subcellular localization of Phactr1. NIH3T3 cells were transfected with Flag-Phactr1 expression plasmid for 4 h. For further 20 h, the cells were cultured under serum-starved conditions (serum−) in the presence of either 10 µM CCG-1423 (+) or vehicle (DMSO) and were then re-stimulated with 10% serum for 15 min (serum+). The cells were stained with anti-DYKDDDDK (Flag) antibody and Hoechst 33258 (upper panel). Bar  = 20 µm. The images were quantified as described in Materials and Methods: nuclear-specific localization (N), diffuse distribution in the nucleus and the cytoplasm (NC), and cytoplasmic localization (C) (lower panel). Asterisks indicate differences from the values under serum re-stimulated conditions without CCG-1423 in the respective localization categories (*P  = 0.0002 and **P  = 0.0002).

**Figure 7:**
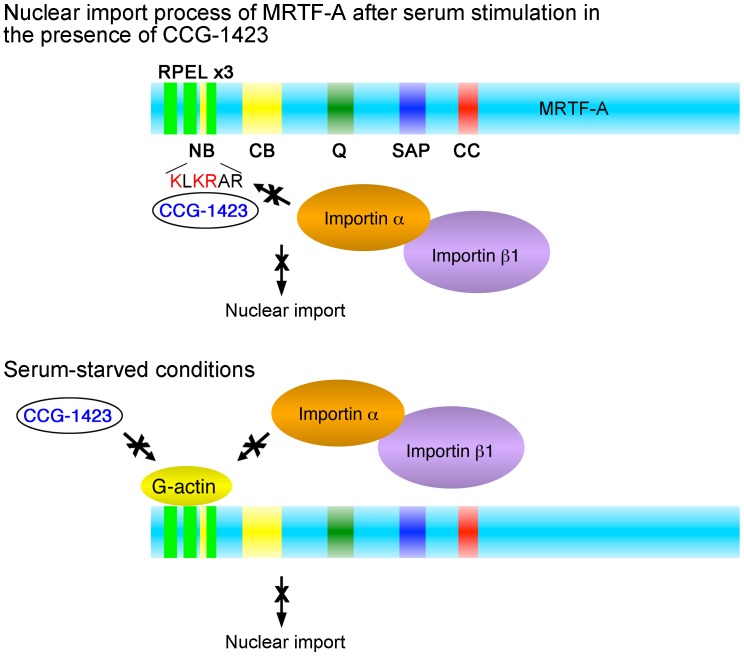
Summary of the inhibitory effect of CCG-1423 on the importin α/β1-mediated nuclear import of MRTF-A. When the G-actin pool is depleted by extracellular stimuli, MRTF-A is imported into the nucleus under mediation by importin α/β1. However, in the presence of CCG-1423, this process is competitively inhibited because CCG-1423 binds to NB and masks the binding site for importin α/β1. The basic amino acids indicated with red letters play a critical role in CCG-1423 binding to NB (upper panel). In contrast, in serum-starved cells, MRTF-A forms a complex with G-actin under mediation by RPEL motifs. Thus, both CCG-1423 and importin α/β1 are not accessible to NB because the binding affinities of CCG-1423 and importin α/β1 to MRTF-A are weaker than those of G-actin to MRTF-A (lower panel). Because MRTF-A associated with G-actin exhibits a low binding affinity to CCG-1423, G-actin-free MRTF-A is a more suitable CCG-1423 target protein. The abbreviations used are as follows: NB, N-terminal basic domain; CB, central basic domain; SAP, SAP domain; CC, coiled-coil domain.

To further address the effects of actin dynamics on MRTF-A binding to CCG-1423 Sepharose, we performed the CCG-1423 binding assay using whole cell extracts from NIH3T3 cells expressing Flag-MRTF-A cultured under different conditions where either cellular F-actin or G-actin levels increased (Figure 4). The effects of Jasp and LatB on cellular F-actin levels in NIH3T3 cells cultured under serum-starved or serum-stimulated conditions were shown (Figure 4A). Treatment with Jasp increased F-actin staining. In contrast, treatment with LatB markedly decreased F-actin staining. Significant binding of Flag-MRTF-A to CCG-1423 Sepharose was detected only in the cell extracts from F-actin-rich culture conditions (Figure 4B, middle panel). These binding properties coincided well with the results of in vitro binding assays shown in Figure 3C. The competitive inhibitory effect of free CCG-1423 was also observed in the CCG-1423 binding assay using whole cell extracts (Figure 4B, lower panel).

### Binding Specificity of CCG-1423

We then investigated whether CCG-1423 binds specifically to NLS of MRTF-A. It has been reported that the nuclear import of Nrf2, a transcription factor essential for antioxidant response element-mediated gene expression, is mediated by three distinct basic amino acid-rich NLSs (Figure 5A) and importin α/β1 [28]. We confirmed that Nrf2 forms a complex with importin α/β1 (Figure 5B). Although the sequences of Nrf2 NLSs are rich in basic amino acids (Figure 5A), significant binding of Nrf2 to CCG-1423 Sepharose was not observed (Figure 5C). Furthermore, the pull-down assay showed that importin α/β1 did not bind to CCG-1423 Sepharose (Figure 5D). These results suggest that CCG-1423 does not bind to any protein with a basic amino acid-rich NLS.

We addressed the binding specificity of CCG-1423 to other Mycd family members (MRTF-B and Mycd) and one of other RPEL containing proteins (Phactr1). Figure 6A shows the sequences of NLSs of Mycd family members and Phactr1. The sequence of NLS of the Mycd family (NB) is conserved among all members from different species and is located between the second and third RPEL motifs [17]. The conserved amino acids of NLS across Mycd family members and Phactr1 were highlighted. Similarly, Phactr1 C-terminal NLS is located between the third and fourth RPEL motifs [20]. We performed a pull-down assay using CCG-1423 Sepharose to examine the binding of respective RPEL-containing proteins to CCG-1423 (Figure 6B). In these assays, in vitro- translated Flag-tagged proteins were purified using an anti-Flag M2 affinity gel and were used as inputs. These analyses revealed that MRTF-B, Mycd, and Phactr1 bound to CCG-1423 Sepharose. Bindings of Flag-MRTF-B and Phactr1 to CCG-1423 Sepharose were also observed in the binding assay using whole cell extracts (Figure S2). The binding of mutant MRTF-B protein with mutation in NB (MRTF-B NBmut) to CCG-1423 Sepharose severely reduced, suggesting that CCG-1423 also binds to MRTF-B under mediation by NB (Figure S3A). We then examined the effect of CCG-1423 on the subcellular localization of exogenously expressed Flag-MRTF-B (Figure S3B) and Flag-Phactr1 (Figure 6C) in NIH3T3 cells under serum-starved and serum-stimulated conditions. In almost all of the cells expressing Flag-MRTF-B under serum-starved conditions, the protein was primarily observed in the cytoplasm. In contrast, in a large proportion (51.7±1.0%) of serum-stimulated cells, Flag-MRTF-B protein accumulated primarily in the nucleus. CCG-1423 treatment significantly reduced (25.1±0.1%) the proportion of cells showing the nuclear accumulation of the protein and increased (48.3±4.0%) the proportion of cells showing the cytoplasmic localization of the protein. (Figure S3B). Similarly, in almost all of the cells expressing Flag-Phactr1 under serum-starved conditions, the protein was located entirely in the cytoplasm. However, in most of the cells under serum-stimulated conditions, the protein was evenly distributed in the cytoplasm and nucleus. CCG-1423 treatment reduced (66.2±2.0%) the proportion of such cells and increased (33.8±2.0%) the proportion of cells showing the cytoplasmic localization of the protein. These results suggest that CCG-1423 inhibits the serum-induced nuclear import of MRTF-B and Phactr1. However, CCG-1423 did not affect the subcellular localization of constitutively nuclear Mycd (data not shown).

## Discussion

CCG-1423, which was originally identified as an inhibitor of RhoA signaling [21], is thought to be the MRTF-A inhibitor because CCG-1423 reduces cell growth and migration and blocks the nuclear accumulation of MRTF-A [22], [23]. However, the mode of inhibitory action is yet to be determined. In this study, we addressed our hypothesis that CCG-1423 directly inhibits MRTF-A binding to importin α/β1. Our novel findings are as follows: (1) CCG-1423 inhibits the interaction between MRTF-A and importin α/β1 but not G-actin binding to MRTF-A, (2) A pull-down assay using CCG-1423 Sepharose revealed direct and specific binding of CCG-1423 to MRTF-A. Furthermore, the functional NLS of MRTF-A (NB) is the binding site for CCG-1423, (3) In the presence of G-actin, MRTF-A preferentially forms a complex with G-actin rather than CCG-1423 because of its high binding affinity for G-actin, indicating competitive binding of G-actin and CCG-1423 to the N-terminal region of MRTF-A containing three RPEL motifs and NB, but it remains elusive whether or not all basic amino acid rich NLS bind to CCG-1423, and (4) CCG-1423 is expected to specifically bind to the NLSs of RPEL-containing proteins such as Mycd family members and Phactr1. These results suggest that CCG-1423 prevents the interaction between MRTF-A and importin α/β1 by masking NB, resulting in inhibition of the nuclear import of MRTF-A and that G-actin-free MRTF-A is the more likely CCG-1423 target protein. These molecular mechanisms are schematically summarized in Figure 7. A similar inhibitory action is expected to be applicable to the interaction between MRTF-B or Phactr1 and importin α/β1.

CCG-1423 inhibits the interaction between MRTF-A and importin α/β1 (Figure 1A). We demonstrated that CCG-1423 binds directly and specifically to MRTF-A under mediation by NB (Figure 3B) and that the basic amino acids in the NB sequence (Figure 7) play a critical role in CCG-1423 binding to NB. Because the sequences of NBs of Mycd family members are identical (Figure 6A), CCG-1423 is expected to bind to each of the NB sequences of MRTF-B and Mycd. Actually, we demonstrated that CCG-1423 binds to MRTF-B under mediation by NB (Figure S3A). CCG-1423 also binds to Phactr1. Although we have not identified the binding site, CCG-1423 is expected to bind to Phactr1 C-terminal NLS (KREIKRR) because this NLS is also located between two RPEL motifs. However, CCG-1423 does not simply recognize a cluster of basic amino acids because CCG-1423 scarcely binds to Nrf2, in which three distinct basic amino acid-rich NLSs are present (Figure 5). CCG-1423 has a strict affinity for a specific sequence and/or tertiary protein structure. Further study is necessary to reveal the binding specificity of CCG-1423. Another possibility is that CCG-1423 inhibits the function of importin α/β1 in the nuclear import machinery. However, this possibility is less likely because importin α/β1 does not bind to CCG-1423 Sepharose (Figure 5D).

We demonstrated that G-actin-free MRTF-A is the more likely CCG-1423 target protein (Figures 3C and 4). These results suggest that CCG-1423 immediately binds to MRTF-A under conditions where Rho-activation induces rapid depletion of the G-actin pool and prevents the interaction between MRTF-A and importin α/β1 in living cells. In resting cells, MRTF-A forms a stable complex with G-actin, and this complex formation significantly suppresses the interaction between MRTF-A/B and importin α/β1 [17], [19]. Thus, CCG-1423 is effective only under conditions where the G-actin pool is depleted.

The Larsen group has most recently reported that CCG-1423 binds specifically to an unknown 24-kD protein in PC-3 cell lysates using tag-free photoaffinity probes [29], suggesting that another target of CCG-1423 exists. Scarce information is currently available about this protein; therefore, future study is required to clarify its function. In their study, high molecular weight proteins (>140 kD) were not detected. This result would be explained by the complex formation between MRTF-A and G-actin; CCG-1423 is less likely to bind to MRTF-A associated with G-actin. The nuclear accumulation of MRTF-A occurs transiently just after serum stimulation and thereafter nuclear MRTF-A is gradually exported to the cytoplasm. Re-stimulation with fresh serum induces the nuclear accumulation of MRTF-A again (Figure S4). In the cytoplasm, MRTF-A forms a stable complex with G-actin. The Larsen group probably used the proliferating PC-3 cell lysates. However, for the reasons stated above, they could not detect MRTF-A/B.

Our present findings provide a new strategy for anti-EMT drug discovery by focusing on the nuclear import of MRTF-A. Immobilization of small molecules on Sepharose or microplates using a photoaffinity reaction is an effective method for detection of small molecule–protein interactions. This system using CCG-1423 as the leading compound would be a useful tool for anti-EMT drug screening because non-specific binding to CCG-1423 Sepharose was not detected in our study (Figures 3 and 5). Furthermore, we are currently working to determine whether a high-throughput screening system could be established using a series of CCG-1423-related compounds immobilized on microarrays and purified MRTF-A protein with fluorescent tag.

In conclusion, CCG-1423 binds specifically to MRTF-A under mediation by the NB, resulting in inhibition of the interaction between MRTF-A and importin α/β1. However, this inhibitory action of CCG-1423 is restricted to the conditions where the G-actin pool is depleted. A similar inhibitory action is expected be applicable to the interaction between MRTF-B or Phactr1 and importin α/β1.

## Supporting Information

Figure S1 **Effects of CCG-1423 on the subcellular localization of MRTF-A.** (A) NIH3T3 cells were transfected with Flag-MRTF-A expression plasmid for 4 h. For a further 20 h, the cells were cultured under serum-starved conditions (serum−) either in the presence of 10 µM CCG-1423 (+) or vehicle, and then were re-stimulated with 10% serum for 15 minutes (serum+). The cells were stained with anti-DYKDDDDK (Flag) antibody (red) and Hoechst 33258 (blue). Representative images are shown (n  = 3, 100–150 cells/condition in each experiment). Bar  = 20 µm. (B) The images were quantified as described in Materials and Methods: nuclear-specific localization (N), diffuse distribution in the nucleus and the cytoplasm (NC), and cytoplasmic localization (C). Asterisks indicate differences from the values under serum re-stimulated conditions without CCG-1423 in the respective localization categories (*P  = 2.138x10^−6^, **P  = 0.0007, and ***P  = 0.0093). (C) Monitoring the activation of SRF-mediated transcription. NIH3T3 cells were transfected with 500 ng of SM22P-luc, 300 ng of pSVβ-gal, and 200 ng of the expression plasmid for Flag-MRTF-A. The culture conditions are described in Materials and Methods. The luciferase activity without serum re-stimulation was set at 100. Each value represents the means ± s.e.ms of results from three independent experiments. Asterisk indicates difference from the value under serum re-stimulated conditions without CCG-1423 (P  = 0.0022). (D and E) NIH3T3 cells were cultured under the same conditions as described earlier. The cells were stained with anti-MRTF-A antibody (red) and Hoechst 33258 (blue), and the images were quantified as described earlier. Representative images are shown (n  = 3, 100–200 cells/condition in each experiment). Bar  = 20 µm. Asterisks indicate differences from the values under the conditions as described earlier (*P  = 0.0004, **P  = 0.0001, and ***P  = 0.044).
(TIF)

Figure S2 **Binding assays of MRTF-B and Phactr1 to CCG-1423 Sepharose using NIH3T3 cell whole extracts.** Whole cell extracts containing Jasp (Jasp+) were prepared from serum-stimulated NIH3T3 cells expressing each of Flag-MRTF-B and Flag-Phactr1. Brief explanations of the respective whole cell extracts are given in the upper panel: WE1 from Flag-MRTF-B-expressing cells and WE2 from Flag-Phactr1-expressing cells. The details of whole cell extract preparation are described in Materials and Methods. These whole cell extracts were subjected to pull-down assay using CCG-Sepharose (CCG) or control Sepharose (cntl). The proteins bound to CCG-Sepharose or control Sepharose were analyzed by IB with anti-Flag antibody.
(TIF)

Figure S3 **Binding property of CCG-1423 to MRTF-B and the effects of CCG-1423 on the subcellular localization of MRTF-B.** (A) Examination of the binding of purified Flag-MRTF-B proteins [wild-type (wt) and NBmut] to CCG-1423 Sepharose. An MRTF-B NBmut protein carries a mutation in NB, in which the NB sequence KLKRAR was mutated to ALAAAR. The pull-down assays were performed as described in the legend for Figure 3. (B) Effects of CCG-1423 on the subcellular localization of MRTF-B. NIH3T3 cells were transfected with Flag-MRTF-B expression plasmid under serum-stimulated conditions for 4 h. The cells were cultured under serum-starved conditions (serum−) in the presence of either 10 µM CCG-1423 (+) or vehicle (DMSO) for further 20 h and were then re-stimulated with 10% serum for 15 min (serum+). The cells were stained with anti-DYKDDDDK (Flag) antibody and Hoechst 33258 (upper panel). Bar  = 20 µm. The images were quantified as described in Materials and Methods: nuclear-specific localization (N), diffuse distribution in the nucleus and the cytoplasm (NC), and cytoplasmic localization (C) (lower panel). Asterisks indicate differences from the values under serum re-stimulated conditions without CCG-1423 in the respective localization categories (*P  = 4.024×10^−6^ and **P  = 0.0015).
(TIF)

Figure S4 **Gradual nuclear export of MRTF-A under serum-stimulated conditions.** NIH3T3 cells were cultured under serum-starved conditions for 20 h (serum−) and were then re-stimulated with 10% serum (serum+) for 15 min and 24 h, respectively. Twenty-four hours later, the cells were re-stimulated with fresh serum for 15 min (serum+24 h/serum+15 min). The cells were stained with anti-MRTF-A antibody (red). Bar  = 20 µm.
(TIF)
