# Supplementary figures and images for: RPEL Proteins Are the Molecular Targets for CCG-1423, an Inhibitor of Rho Signaling
Source: PLoS One. 2014 Feb 18;9(2):e89016. doi: 10.1371/journal.pone.0089016 (PMC3928398; doi:10.1371/journal.pone.0089016)

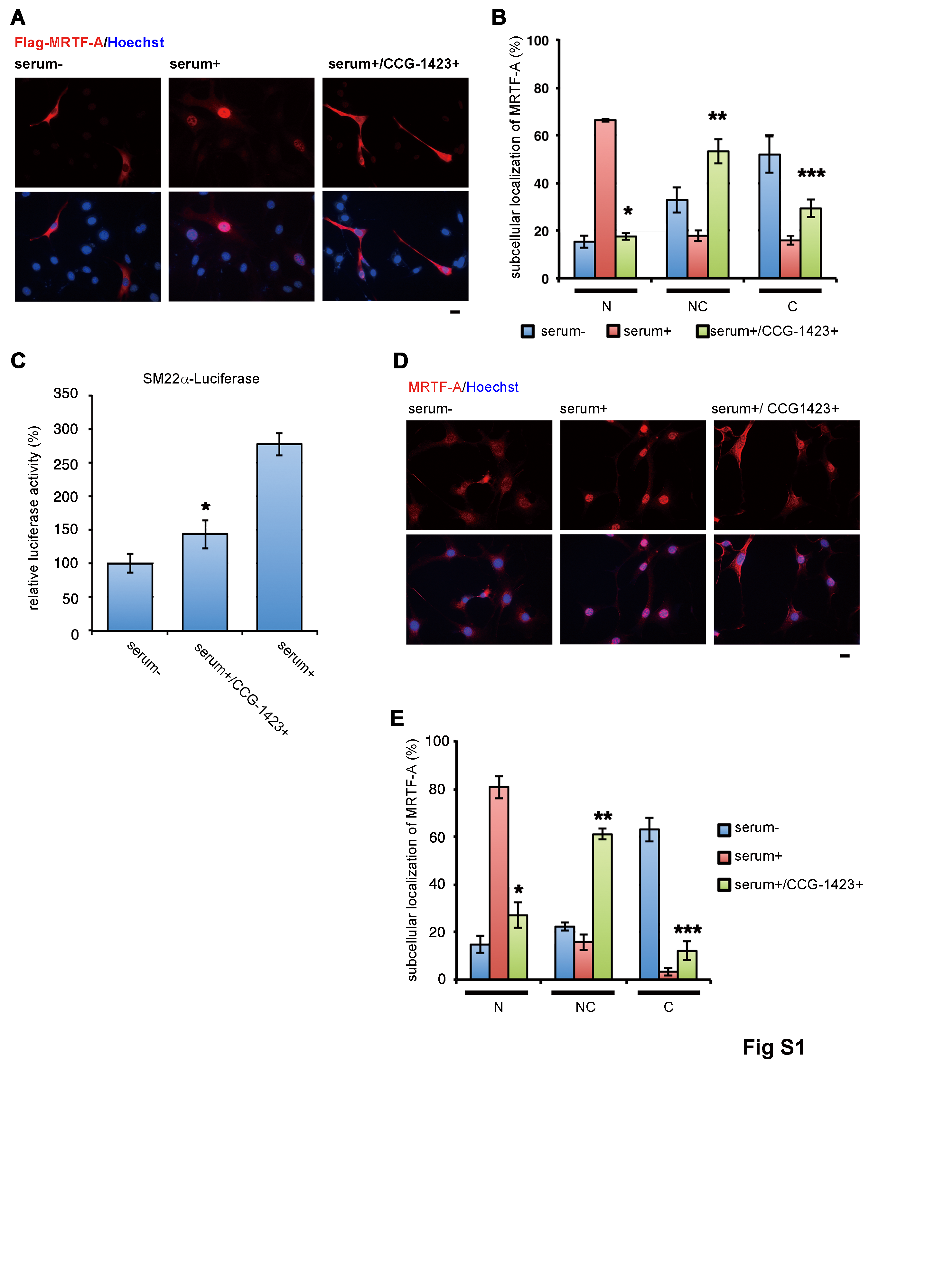

Supplement: Figure S1 — Effects of CCG-1423 on the subcellular localization of MRTF-A. (A) NIH3T3 cells were transfected with Flag-MRTF-A expression plasmid for 4 h. For a further 20 h, the cells were cultured under serum-starved conditions (serum−) either in the presence of 10 µM CCG-1423 (+) or vehicle, and then were re-stimulated with 10% serum for 15 minutes (serum+). The cells were stained with anti-DYKDDDDK (Flag) antibody (red) and Hoechst 33258 (blue). Representative images are shown (n = 3, 100–150 cells/condition in each experiment). Bar = 20 µm. (B) The images were quantified as described in Materials and Methods: nuclear-specific localization (N), diffuse distribution in the nucleus and the cytoplasm (NC), and cytoplasmic localization (C). Asterisks indicate differences from the values under serum re-stimulated conditions without CCG-1423 in the respective localization categories (*P = 2.138x10^−6^, **P = 0.0007, and ***P = 0.0093). (C) Monitoring the activation of SRF-mediated transcription. NIH3T3 cells were transfected with 500 ng of SM22P-luc, 300 ng of pSVβ-gal, and 200 ng of the expression plasmid for Flag-MRTF-A. The culture conditions are described in Materials and Methods. The luciferase activity without serum re-stimulation was set at 100. Each value represents the means ± s.e.ms of results from three independent experiments. Asterisk indicates difference from the value under serum re-stimulated conditions without CCG-1423 (P = 0.0022). (D and E) NIH3T3 cells were cultured under the same conditions as described earlier. The cells were stained with anti-MRTF-A antibody (red) and Hoechst 33258 (blue), and the images were quantified as described earlier. Representative images are shown (n = 3, 100–200 cells/condition in each experiment). Bar = 20 µm. Asterisks indicate differences from the values under the conditions as described earlier (*P = 0.0004, **P = 0.0001, and ***P = 0.044). (TIF) [file pone.0089016.s001.tif]

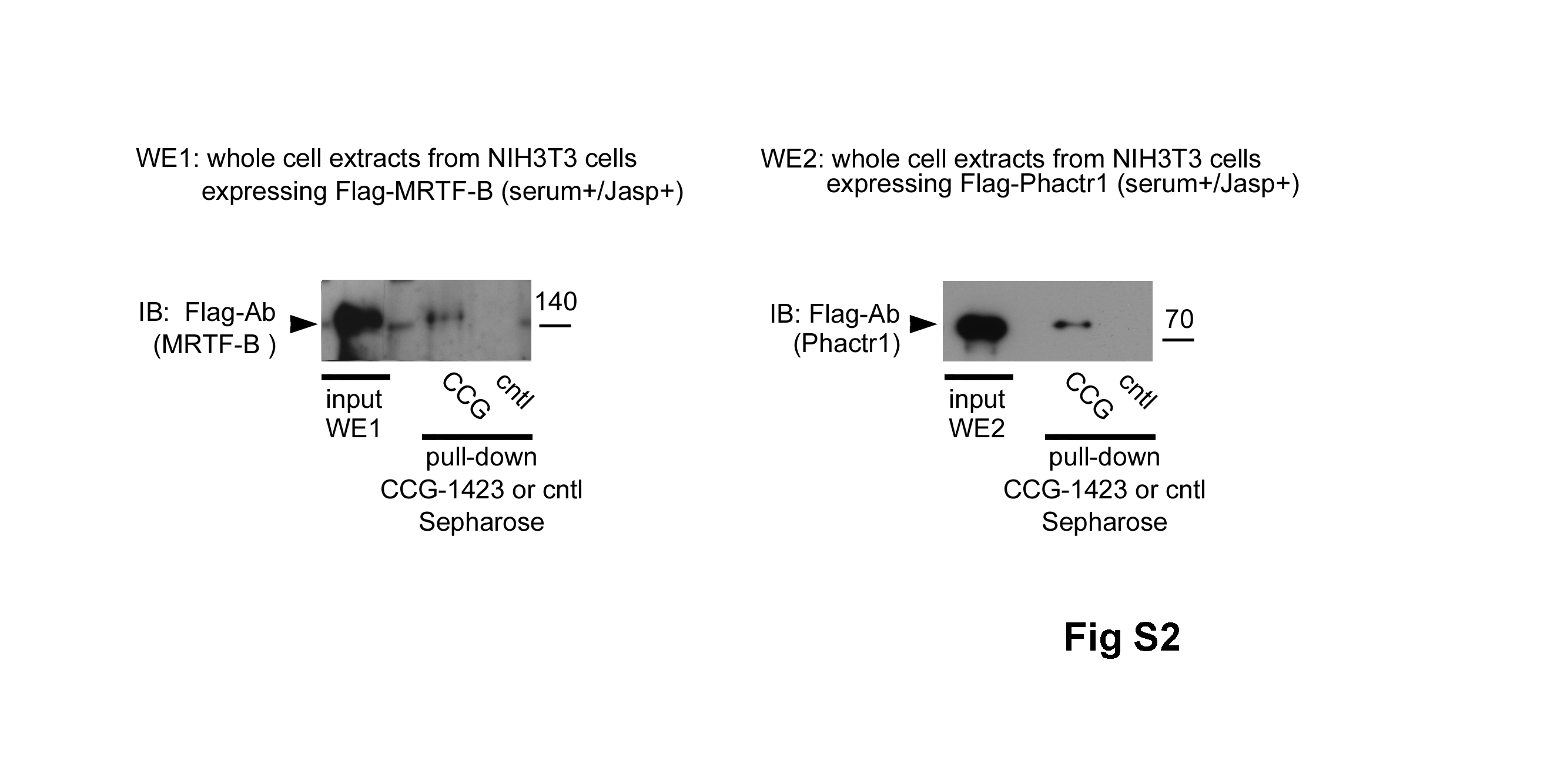

Supplement: Figure S2 — Binding assays of MRTF-B and Phactr1 to CCG-1423 Sepharose using NIH3T3 cell whole extracts. Whole cell extracts containing Jasp (Jasp+) were prepared from serum-stimulated NIH3T3 cells expressing each of Flag-MRTF-B and Flag-Phactr1. Brief explanations of the respective whole cell extracts are given in the upper panel: WE1 from Flag-MRTF-B-expressing cells and WE2 from Flag-Phactr1-expressing cells. The details of whole cell extract preparation are described in Materials and Methods. These whole cell extracts were subjected to pull-down assay using CCG-Sepharose (CCG) or control Sepharose (cntl). The proteins bound to CCG-Sepharose or control Sepharose were analyzed by IB with anti-Flag antibody. (TIF) [file pone.0089016.s002.tif]

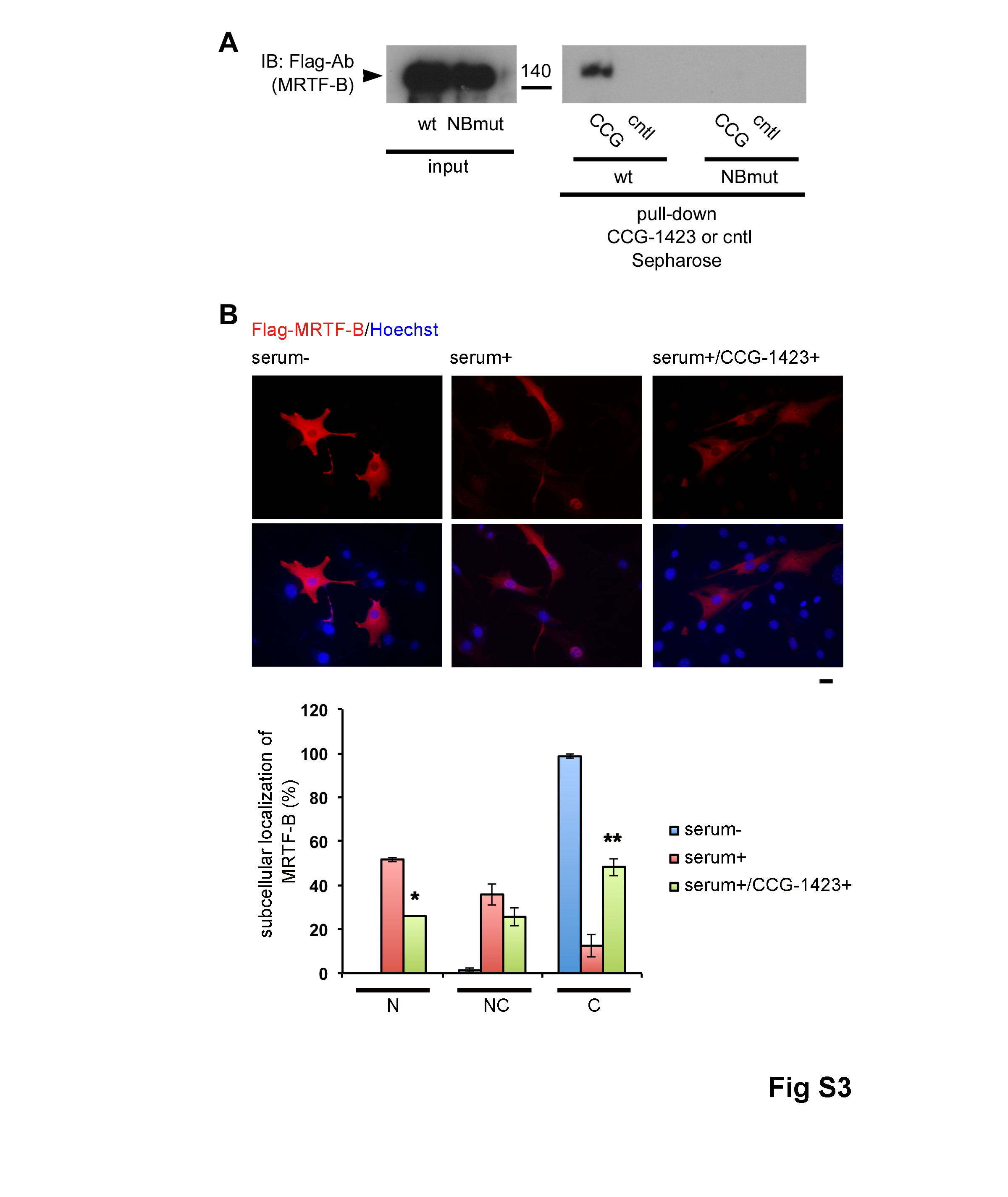

Supplement: Figure S3 — Binding property of CCG-1423 to MRTF-B and the effects of CCG-1423 on the subcellular localization of MRTF-B. (A) Examination of the binding of purified Flag-MRTF-B proteins [wild-type (wt) and NBmut] to CCG-1423 Sepharose. An MRTF-B NBmut protein carries a mutation in NB, in which the NB sequence KLKRAR was mutated to ALAAAR. The pull-down assays were performed as described in the legend for Figure 3. (B) Effects of CCG-1423 on the subcellular localization of MRTF-B. NIH3T3 cells were transfected with Flag-MRTF-B expression plasmid under serum-stimulated conditions for 4 h. The cells were cultured under serum-starved conditions (serum−) in the presence of either 10 µM CCG-1423 (+) or vehicle (DMSO) for further 20 h and were then re-stimulated with 10% serum for 15 min (serum+). The cells were stained with anti-DYKDDDDK (Flag) antibody and Hoechst 33258 (upper panel). Bar = 20 µm. The images were quantified as described in Materials and Methods: nuclear-specific localization (N), diffuse distribution in the nucleus and the cytoplasm (NC), and cytoplasmic localization (C) (lower panel). Asterisks indicate differences from the values under serum re-stimulated conditions without CCG-1423 in the respective localization categories (*P = 4.024×10^−6^ and **P = 0.0015). (TIF) [file pone.0089016.s003.tif]

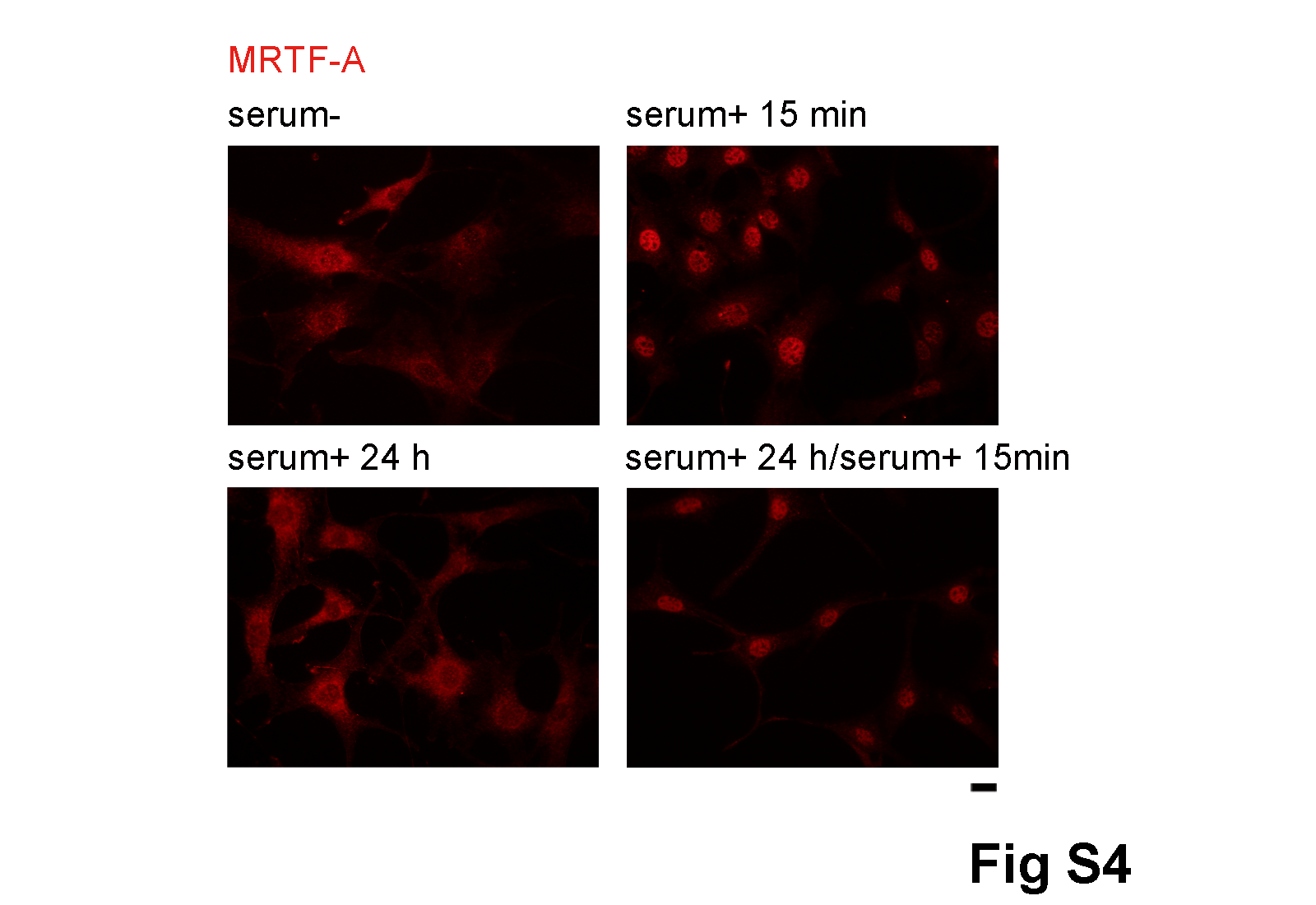

Supplement: Figure S4 — Gradual nuclear export of MRTF-A under serum-stimulated conditions. NIH3T3 cells were cultured under serum-starved conditions for 20 h (serum−) and were then re-stimulated with 10% serum (serum+) for 15 min and 24 h, respectively. Twenty-four hours later, the cells were re-stimulated with fresh serum for 15 min (serum+24 h/serum+15 min). The cells were stained with anti-MRTF-A antibody (red). Bar = 20 µm. (TIF) [file pone.0089016.s004.tif]
